# Phosphorylation of cPLA_2_α at Ser^505^ Is Necessary for Its Translocation to PtdInsP_2_-Enriched Membranes

**DOI:** 10.3390/molecules27072347

**Published:** 2022-04-06

**Authors:** Javier Casas, Jesús Balsinde, María A. Balboa

**Affiliations:** 1 Instituto de Biología y Genética Molecular, Consejo Superior de Investigaciones Científicas (CSIC), 47003 Valladolid, Spain; mbalboa@ibgm.uva.es; 2 Centro de Investigación Biomédica en Red de Diabetes y Enfermedades Metabólicas Asociadas (CIBERDEM), 28029 Madrid, Spain

**Keywords:** cytosolic phospholipase A_2_α, arachidonic acid, membrane translocation, phosphorylation, phosphatidylinositol bisphosphate

## Abstract

Group IVA cytosolic phospholipase A_2_α (cPLA_2_α) is a key enzyme in physiology and pathophysiology because it constitutes a rate-limiting step in the pathway for the generation of pro- and anti-inflammatory eicosanoid lipid mediators. cPLA_2_α activity is tightly regulated by multiple factors, including the intracellular Ca^2+^ concentration, phosphorylation reactions, and cellular phosphatidylinositol (4,5) bisphosphate levels (PtdInsP_2_). In the present work, we demonstrate that phosphorylation of the enzyme at Ser^505^ is an important step for the translocation of the enzyme to PtdInsP_2_–enriched membranes in human cells. Constructs of eGFP-cPLA_2_ mutated in Ser^505^ to Ala (S505A) exhibit a delayed translocation in response to elevated intracellular Ca^2+^, and also in response to increases in intracellular PtdInsP_2_ levels. Conversely, translocation of a phosphorylation mimic mutant (S505E) is fully observed in response to cellular increases in PtdInsP_2_ levels. Collectively, these results suggest that phosphorylation of cPLA_2_α at Ser^505^ is necessary for the enzyme to translocate to internal membranes and mobilize arachidonic acid for eicosanoid synthesis.

## 1. Introduction

The eicosanoids constitute an ample family of bioactive lipids with potent pro- and anti-inflammatory activities. They are not stored in the cells but produced in response to specific cellular stimulation [1,2]. The common precursor of the eicosanoids is arachidonic acid (AA), a fatty acid that is mostly found esterified in the sn-2 position of cellular glycerophospholipids, primarily those containing choline, ethanolamine, and inositol as polar headgroups [3,4,5]. Although the cells possess multiple phospholipase A_2_ (PLA_2_) enzymes potentially capable of liberating AA from membrane phospholipids [6,7,8,9], the major PLA_2_ involved in receptor-mediated AA mobilization is the group IVA PLA_2_, also known as cytosolic PLA_2_α (cPLA_2_α) [10,11].

cPLA_2_α is tightly regulated in cells, not only to finely regulate the amount of AA mobilized for eicosanoid synthesis but also because both of its by-products, free fatty acids and lysophospholipids, could be deleterious to the cells if accumulating at high levels. Increases in the intracellular Ca^2+^ concentration constitute one of the key regulators of cPLA_2_α activity in cells and mediate translocation of the enzyme to a variety of cytoplasmic membranes [10,11]. This is driven by the presence in the enzyme of a calcium-binding domain, or C2 domain. Unlike other PLA_2_ family members, cPLA_2_α does not require Ca^2+^ for enzyme activity, but to dock and penetrate into the membrane interface [12,13,14].

In addition to Ca^2+^, cPLA_2_α is also regulated by intracellular lipids. The C2 domain also has a site for ceramide 1-phosphate, produced by activated ceramide kinase in situ [15,16]. This lipid allosterically activates the enzyme and increases the residence time of the enzyme in membranes [15,16]. cPLA_2_α also binds phosphatidylinositol 4,5-bisphosphate (PtdInsP_2_) via a 4-Lys cluster present in the catalytic domain [17,18]. In vitro, PtdInsP_2_ increases the catalytic activity of the enzyme in a calcium-independent manner, likely by enhancing its capacity to penetrate membranes, especially those enriched in choline phospholipids [17,18]. Mutations in the residues where PtdInsP_2_ binds give rise to an enzyme that, when transfected into cells, manifests a reduced ability to translocate to intracellular membranes and mobilize AA [19,20].

cPLA_2_α can also be phosphorylated in cells at residues Ser^505^, Ser^515^, and Ser^727^, and all of these phosphorylation reactions have been suggested to be involved in the regulation of agonist-induced AA mobilization [10,11,21]. While the functional relevance of cPLA_2_α phosphorylation at Ser^515^ and Ser^727^ may depend on cell type and stimulation conditions, there is general agreement that phosphorylation at Ser^505^ represents a key regulatory event under nearly all cellular conditions examined; thus, it has been the most extensively studied [10,11,21]. In general terms, it appears that the extracellular-regulated kinases p42/p44 are responsible for cPLA_2_α phosphorylation at Ser^505^ in cells of murine origin [22,23,24], and the related kinases p38 and JNK are involved in cells of human origin [25,26,27].

Several lines of evidence have suggested that cPLA_2_α phosphorylation at Ser^505^ is necessary for the enzyme to be fully active in cells; however, the molecular reasons for this still remain elusive [21]. In vitro studies have indicated that phosphorylation of cPLA_2_α at Ser^505^ is not required for activity but for proper binding of the enzyme to membranes in a Ca^2+^-dependent fashion [28].

Previous work from our laboratory has highlighted the importance of intracellular PtdInsP_2_ levels in regulating the physical state of cPLA_2_α at Ca^2+^ levels equaling those present in resting cells [19]. The phosphorylation state of cPLA_2_α does not seem to influence the translocation of the enzyme to model membranes in the presence of high Ca^2+^ [28]. In the present study, we have studied the influence of cPLA_2_α phosphorylation at Ser^505^ on PtdInsP_2_ binding and in the translocation capacity of the enzyme in a cellular scenario. We show that cPLA_2_α phosphorylation at Ser^505^ is necessary for the translocation of the enzyme to membranes and to promote AA release in response to PtdInsP_2_ elevations. These studies provide new insights into the complex regulation of cPLA_2_α, thereby expanding and deepening our knowledge of the cellular mechanisms controlling the production of pro- and anti-inflammatory lipid mediators.

## 2. Results

### 2.1. Role of cPLA_2_α Phosphorylation in Membrane Translocation in Response to Increases in PtdInsP_2_ and Calcium

**Figure 1:**
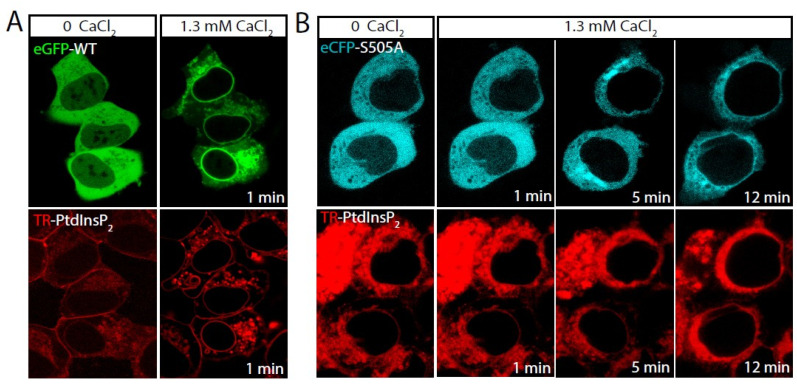
Translocation of eGFP-cPLA_2_α and eCFP-S505A-cPLA_2_α in response to PtdInsP_2_. HEK cells stably transfected with eGFP-cPLA_2_α (**A**) or the mutant eCFP-S505A-cPLA_2_α (**B**) were incubated with TR-PtdInsP_2_/histone in the absence of extracellular Ca^2+^ (labeled as 0 CaCl_2_) for 10 min. Afterward, 1.3 mM CaCl_2_ was added to the medium to restore extracellular calcium levels (labeled as 1.3 mM CaCl_2_). Pictures were taken under the confocal microscope at the indicated time points. Upper panels show the fluorescence from the cPLA_2_α constructs, while lower panels show fluorescence from the TR-PtdInsP_2_.

In previous work, we showed that increasing PtdInsP_2_ levels in intact cells in the absence of a rise in intracellular Ca^2+^ is sufficient to trigger cPLA_2_α activation and attendant AA mobilization [19]. We began the current study by assessing whether such an effect of PtdInsP_2_ requires the enzyme to be phosphorylated at Ser^505^. To this end, cells stably expressing eGFP-cPLA_2_α or the mutant eCFP-S505A-cPLA_2_α were incubated with exogenous PtdsInsP_2_ complexed with a histone shuttle to facilitate entry into the cells. We and others have previously used this technique successfully [19,29,30], which results in cells displaying increased PtdInsP_2_ levels, and no influence on the physical state of cPLA_2_α, as the incubations are carried out in the absence of extracellular Ca^2+^ [19]. The PtdsInsP_2_ contained a fluorescent tag that allowed its monitoring within the cells by confocal microscopy. Once the cells incorporated all the PtdsInsP_2_ (as assessed by the cellular fluorescence associated with it, which remained stable and did not increase further), restoring extracellular Ca^2+^ levels triggered the immediate translocation of cPLA_2_α, i.e., 1 min, to internal membranes (Figure 1A). Importantly, the non-phosphorylatable eCFP-S505A-cPLA_2_α mutant required considerably more time, i.e., 5–12 min, to translocate to perinuclear membranes (Figure 1B).

**Figure 2:**
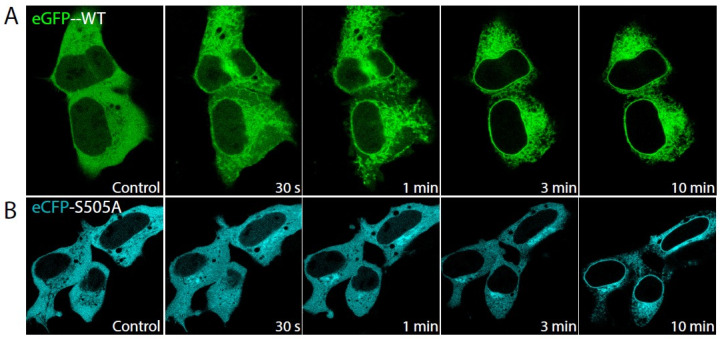
Translocation of eGFP-cPLA_2_α and eCFP-S505A-cPLA_2_α in response to high Ca^2+^ concentrations. Fluorescence from HEK cells stably transfected with eGFP-cPLA_2_α (**A**) or the mutant eCFP-S505A-cPLA_2_α (**B**) was analyzed by confocal microscopy before (Control) or after stimulation with 5 µM ionomycin for the indicated periods of time.

Because PtdInsP_2_ is known to reduce the Ca^2+^ threshold for cPLA_2_α to translocate to membranes in vitro [17,31], in the next series of experiments, we examined the behavior of the enzyme and the S505A mutant under the opposite circumstances, i.e., in the presence of a sustained rise of the intracellular Ca^2+^ level. As shown in Figure 2, cell treatment with 5 µM ionomycin, which raises the intracellular Ca^2+^ concentration up to 4 µM [19,32], the eCFP-S505A-cPLA_2_α translocated to inner membranes after experiencing a marked delay compared with wild type EGFP-cPLA_2_α. The former required approx. 10 min to target the perinuclear membranes (Figure 2B), while the latter was translocated completely to perinuclear membranes within the first 3 min of treatment (Figure 2A). Collectively, these data indicate that phosphorylation of cPLA_2_α at Ser^505^ is a key step for the enzyme to readily translocate to its target membranes even at high intracellular Ca^2+^ levels.

### 2.2. Phosphorylation of cPLA_2_α Is Necessary for AA Release in Response to PtdInsP_2_

**Figure 3:**
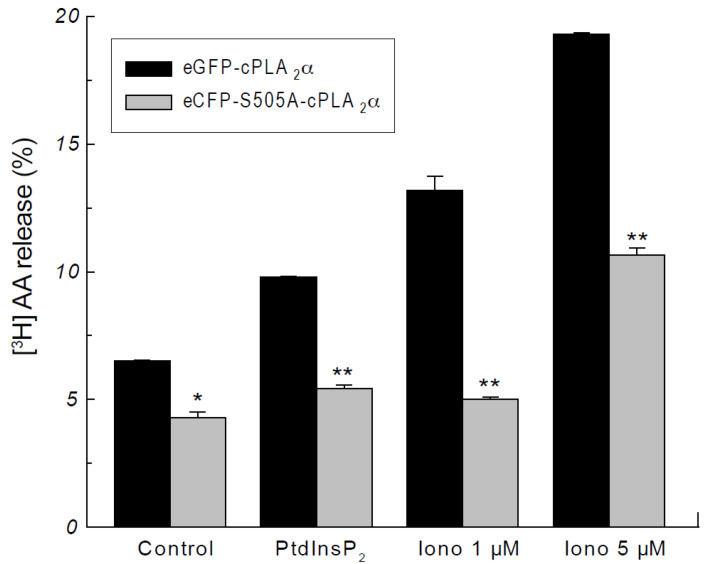
cPLA_2_α phosphorylation is necessary for AA release. HEK cells stably transfected with the construct eGFP-cPLA_2_α (black bars) or eCFP-S505A-cPLA_2_α (gray bars) were prelabeled with [^3^H]AA and treated with vehicle (Control), PtdInsP_2_/histone, 1 µM or 5 µM ionomycin (iono), as indicated. AA release was assessed at different times. Data are shown as means ± standard error of the mean (*n* = 4). * *p* < 0.05, ** *p* < 0.01, significantly different, eCFP-S505A-cPLA_2_α versus eGFP-cPLA_2_α at each condition.

To assess the possible physiological/pathophysiological relevance of the delay in membrane translocation of the eCFP-S505A-cPLA_2_α, we also conducted AA mobilization experiments under identical experimental conditions. The cells were prelabeled with [^3^H]AA, and the release of radiolabeled fatty acid was measured after exposing the cells to PtdInsP_2_ and ionomycin. As shown in Figure 3, [^3^H]AA release in cells transfected with the eCFP-S505A-cPLA_2_α mutant was significantly lower than that of cells transfected with the wild-type enzyme, and this occurred at all conditions tested. Thus, delayed cPLA_2_α translocation results in diminished fatty acid mobilization.

**Figure 4:**
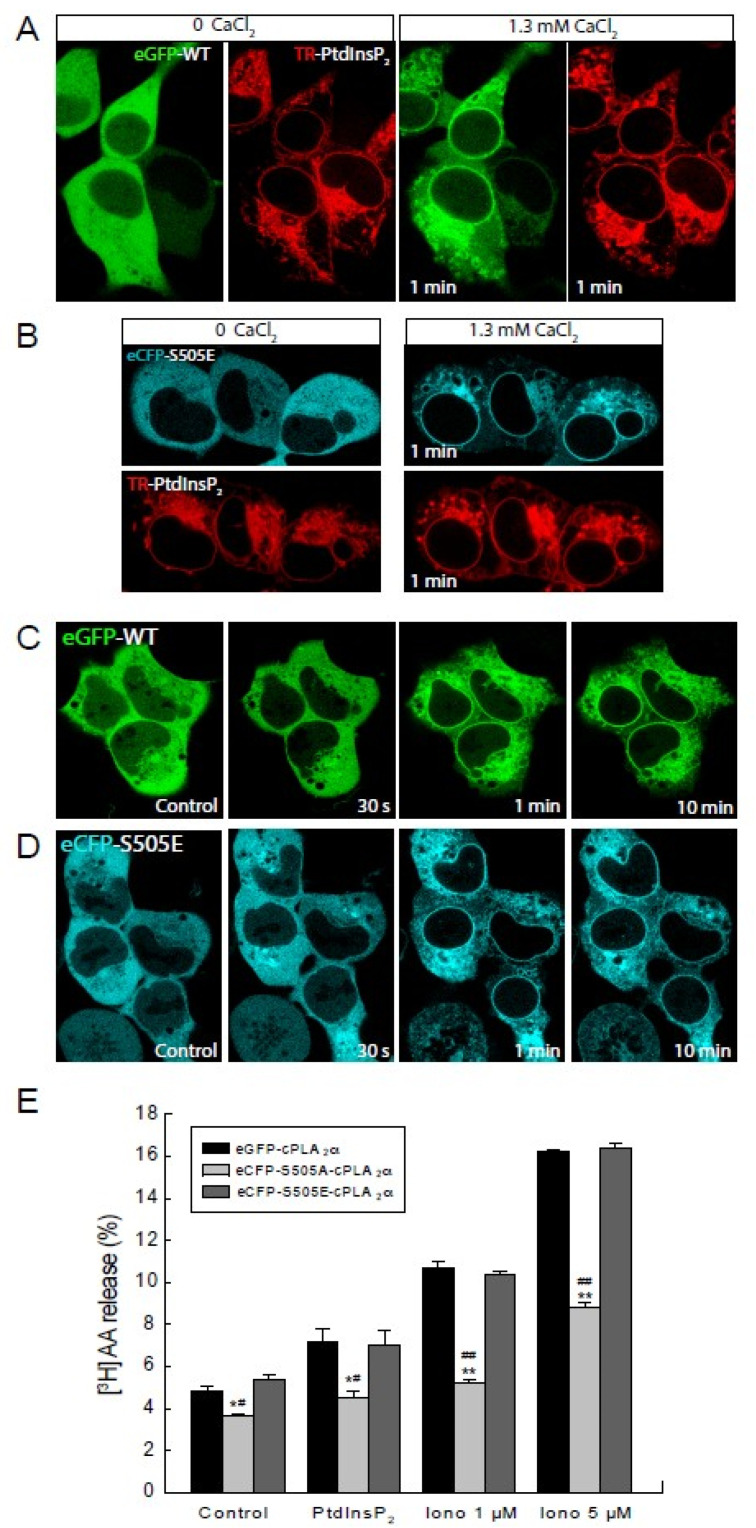
Translocation capabilities and cellular activity of the mutant eCFP-S505E-cPLA_2_α. HEK cells stably transfected with eGFP-cPLA_2_α (**A**,**C**) or the mutant eCFP-S505E-cPLA_2_α (**B**,**D**) were incubated with TR-PtdInsP_2_/histone in the absence of extracellular Ca^2+^ (labeled as 0 CaCl_2_) for 10 min (**A**,**B**). Afterward, 1.3 mM CaCl_2_ was added to the medium to restore extracellular Ca^2+^ levels (labeled as 1.3 mM CaCl_2_) (**A**,**B**). In (**C**,**D**) HEK cells were stimulation with 5 µM ionomycin for the indicated periods of time. Pictures were taken to live cells under the confocal microscope at the indicated time points. (**E**) Cells stably transfected with the construct eGFP-cPLA_2_α (black bars), eCFP-S505A-cPLA_2_α (gray bars), or eCFP-S505E-cPLA_2_α (dark gray bars) were prelabeled with [^3^H]AA and treated with vehicle (Control), PtdInsP_2_/histone, 1 µM or 5 µM ionomycin, as indicated. AA release was assessed at different times. Data are shown as means ± standard error of the mean (*n* = 4). * *p*< 0.05, ** *p* < 0.01, significantly different, eCFP-S505A-cPLA_2_α versus eGFP-cPLA_2_α at each condition. ^#^ *p* < 0.05, ^##^ *p* < 00.1, significantly different, eCFP-S505A-cPLA_2_α versus eCFP-S505E-cPLA_2_α at each condition.

To further characterize the importance of Ser^505^ phosphorylation in cPLA_2_α translocation in response to PtdInsP_2_, mutants were constructed where Ser^505^ was replaced with Glu (S505E), which mimics phosphorylation at that residue [25,28]. The ability of this S505E mutant to translocate in response to PtdInsP_2_ elevations and to sustained increases in intracellular Ca^2+^ was then evaluated, and the results are shown in Figure 4. As expected, the S505E mutant behaved the same as the wild-type mutant (Figure 4A–D). Note that, in common with many other cells in culture [24,33], most of the wild-type cPLA_2_α in the HEK cells is already phosphorylated at Ser^505^. This explains why the S505E mutant behaves the same as the wild-type enzyme. Moreover, cells expressing the S505E mutant manifested an AA release response to either PtdInsP_2_ elevations or ionomycin which was essentially identical to that of cells expressing the wild-type enzyme (Figure 4E). Thus, these data show that cPLA_2_α phosphorylation at Ser^505^ capacitates the enzyme for a full functional response.

## 3. Discussion

The mechanisms responsible for the translocation of cPLA_2_α to cellular membranes in the absence of sustained increases in intracellular calcium have remained a subject of debate [10,11,34]. Early studies utilizing purified cPLA_2_α showed that the binding of the enzyme to vesicles and micelles increased in the presence of PtdInsP_2_, resulting in enhanced activity even at nanomolar Ca^2+^ levels [17,18,31]. Studies in intact cells have also provided evidence that increased PtdInsP_2_ levels in cells can sustain cPLA_2_α activation and attendant AA mobilization at Ca^2+^ levels equaling those of resting cells [34,35]. Finally, a four-Lys cluster was described in the enzyme, which binds PtdInsP_2_ tightly and may help regulate the cellular location of the enzyme under stimulatory conditions [19,20]. In this work, we extend our knowledge of PtdInsP_2_ regulation of cPLA_2_α by showing for the first time that phosphorylation of the enzyme at Ser^505^ is necessary for the full regulatory effect of PtdInsP_2_ to take place. Thus, these results establish a hitherto unrecognized link between two major mechanisms of cPLA_2_α regulation, namely PtdInsP_2_ and Ser^505^ phosphorylation.

While phosphorylation of cPLA_2_α at Ser^505^ has been recognized for a long time, its full physiological significance remains unclear. Reasons for this include the finding that in resting cells, most of the cPLA_2_α is already phosphorylated at Ser^505^ and that the specific activity of the non-phosphorylated enzyme differs little from that of the phosphorylated one [24,33,36]. Our data support the view that PtdInsP_2_ may help the cPLA_2_α to achieve the appropriate conformation for optimal interaction of the enzyme with its target membrane, in agreement with previous observations [28]. cPLA_2_α is a rather ‘promiscuous’ enzyme, being able to translocate to different membranes depending on cell type and stimulatory conditions [11,37]. Whether PtdInsP_2_ regulates the translocation of cPLA_2_α to all kinds of intracellular membranes or its regulatory function is limited to targeting the enzyme to specific membranes is unknown at present. It is also interesting to note that the kinases involved in phosphorylating cPLA_2_α at Ser^505^ under activation conditions appear to greatly depend on cell type and stimulation conditions [22,23,24,25,26,27]. Whether these differences are due to species-specific features or reflect distinctive regulatory attributes of the enzyme is also unknown. Future work in the laboratory will be aimed to investigate whether the PtdInsP_2_ effects on enzyme translocation are related to the involvement of a specific kinase or intracellular membrane.

Stimulatory cell conditions of physiological/pathophysiological relevance do not lead to high calcium concentrations inside the cell. Rather, receptor-mediated activation promotes low and transient increases in Ca^2+^ concentration, which on many occasions lead to cPLA_2_α activation [10,11,12]. Thus, it seems necessary to define the factors that regulate the translocation of cPLA_2_α to membranes under physiological Ca^2+^ conditions. Several lines of evidence have suggested that the enzyme behaves differently depending on Ca^2+^ availability. Under high Ca^2+^ concentrations (>1 µM), the C2 domain of the enzyme is fully active and can drive translocation of the cPLA_2_α to membranes without any other requirement [12,34]. On the contrary, under physiologically relevant Ca^2+^ conditions (up to 400 nM), multiple regulatory components may have to be set into motion to achieve full translocation of the enzyme. We have shown here that cPLA_2_α phosphorylation at Ser^505^ is one of these components.

The finding that the non-phosphorylatable mutant S505A shows a reduced ability to translocate to membranes in response to PtdInsP_2_ elevations suggests that, in the absence of phosphorylation, cPLA_2_α is not capable of binding productively to PtdInsP_2_, probably because the affinity for the phospholipid is decreased. Alternatively, it is also possible that the S505A mutant requires higher Ca^2+^ levels to translocate to the membrane, even in the presence of PtdInsP_2_. The experiments conducted with the phosphorylation mimetic mutant S505E further support the idea that cPLA_2_α phosphorylation at Ser^505^ is required for the enzyme to recognize and respond to PtdInsP_2_ elevations optimally.

Overall, the findings described here demonstrate that cPLA_2_α has multiple mechanisms to circumvent its necessity for high Ca^2+^ concentrations to translocate to membranes and that those mechanisms interact with each other. Moreover, the diminished translocation ability of the nonphosphorylated enzyme in response to PtdInsP_2_ elevations (at least 11 min delay compared with the wild-type enzyme) underscores the importance of these interactions for cPLA_2_α to display full biological activity.

## 4. Materials and Methods

### 4.1. Plasmids

The plasmid eGFP-cPLA_2_α has been described elsewhere [19,32]. For the construction of the eCFP-S505A-cPLA_2_α, the eGFP was substituted in the plasmid eGFP-cPLA_2_α by the eCFP by using the restriction enzymes Agel and BsrGI. Subsequently, Ser^505^ was replaced with Ala (S505A) by using the QuikChange XL Site-Directed Mutagenesis kit (Stratagene, San Diego, CA, USA), and the oligonucleotides 5′-CAATACATCTTATCCATGG- CGCCTTTGAGTGACTT-3′ (forward) and 5′-GCAAAGTCACTCAAAGGCGCCAGTGGATAAGATGTA-3′ (reverse). For the mutagenesis of Ser505 to Glu (S505E) the oligonucleotides used were: 5′-GAATCTCAATACATCTTATCCACTGGAGCCTTTGAGTGACTTTGC-3′ (forward) and 5′-GCAAAGTCACTCAAAGGCTCCAGTGGATAAGATGTATTGAGATTC-3x (reverse). Mutagenesis was confirmed by sequencing.

### 4.2. Cells

HEK cells were cultured in Dulbecco’s Modified Essential Medium (Gibco, Carlsbad, CA, USA) supplemented with 2 mM glutamine, 10% fetal calf serum, 100 U/mL penicillin, and 100 µg/mL streptomycin at 37 °C in a 5% CO_2_ humidified incubator. Cells were passaged twice a week by trypsinization. Cells (40–70% confluence) were transfected with 1 µg plasmid/mL using Lipofectamine Plus^TM^ (Invitrogen, Carlsbad, CA, USA), following the manufacturer’s instructions. For stably transfected cells, 1 mg/mL G418 was used for selection and subsequent passages.

### 4.3. Lipid Preparation

PtdInsP_2_ was added to the cells as previously described [19,30]. Briefly, 2 µg of phospholipid was mixed with 2 µL of the carrier (histone, 0.5 mM), resuspended in Hank’s balanced salt solution containing 10 mM HEPES, sonicated in a water bath for 2 min, and allowed to rest at 37 °C for 10 min before use. The final concentration of PtdInsP_2_ in the incubation media was 5.7 µM.

### 4.4. Confocal Microscopy

The cells were seeded on glass-bottom culture dishes (MatTek Corp., Ashland, MA, USA) and allowed to adhere for 24 h. The medium was then replaced by Hanks’ buffered saline containing 10 mM HEPES and 1.3 mM CaCl_2_. For some experiments, cells were incubated without CaCl_2_, which was added back when needed. Fluorescence was monitored by confocal microscopy using a Bio-Rad Radiance 2100 laser-scanning system coupled to a Nikon TE-2000U with a termostatized chamber (Warner Instruments, Holliston, MA, USA). The objective was CFI Plan Apo 60X, 1.4 numerical aperture, and oil immersion. The fluorescence of eCFP was monitored at 457 nm argon excitation using the combination of a long pass barrier filter HQ470LP and a short pass filter HQ520SP. The fluorescence of eGFP was monitored at 488 nm Argon excitation using the combination of a long pass filter HQ500LP and a short pass filter HQ560SP. The Alexa-Fluo 594 fluorescence was monitored at 543 nm HeNe excitation using a long band pass filter HQ570LP. Red fluorescence from BODYPI-TRx was monitored at 543 nm HeNe laser excitation using an HQ590/570 long band pass blocking filter.

### 4.5. AA Release

The cells were labeled with 0.5 µCi/mL [^3^H]AA (sp. act. 200 Ci/mmol; HartBiomédica, Madrid, Spain) for 18 h. Afterward, they were washed extensively and overlaid with 0.5 mL of serum-free medium supplemented with 0.5 mg/mL albumin and treated with 5 µM thimerosal for 15 min to block fatty acid reacylation [38,39]. The cells were then stimulated for 60 min. Supernatants were removed, and cell monolayers were overlaid with ice-cold phosphate buffer containing 0.05% Triton X-100 and scraped. Total lipids from supernatants and cells were extracted according to Bligh and Dyer [40]. After extraction, lipids were separated by thin-layer chromatography using the system n-hexane/diethyl ether/acetic acid (70:30:1 by volume) [41]. Spots corresponding to free AA and phospholipid were scraped, and radioactivity was quantified by liquid scintillation counting.

## 5. Conclusions

cPLA_2_α-mediated production of bioactive lipid mediators represents a key event in the execution of physiological and pathophysiological responses to external stimuli. This study has focused on the complex interactions that govern cPLA_2_α translocation to membranes and the multiple factors that may be involved in its regulation. Specifically, we have described the interconnected role of two of these factors, phosphorylation of the enzyme at Ser^505^ and cellular PtdInsP_2_ levels. Both of them seem to work together to promote membrane translocation and activation of cPLA_2_α under low intracellular Ca^2+^ levels. Together, the studies described here represent a relevant working model to further understand the intricacies of the cellular regulation of cPLA_2_α and the molecular mechanisms underlying it.

## Data Availability

Data are contained within the article.
